# NT‐proBNP and BNP Testing in Pulmonary Arterial Hypertension: Point‐of‐Care and Remote Monitoring

**DOI:** 10.1111/resp.70087

**Published:** 2025-07-03

**Authors:** Charlotte Durrington, Christian Battersby, Laura Holt, Alexandra Fairman, Scarlett Strickland, Thomas Salisbury, Helena A. Turton, Lisa Watson, Ian Smith, Stefan Roman, Jenna Ablott, Felicity Hitchcock, Chloe Roddis, Eleanor Oakes, Heather Wilshaw, Iain Woodrow, Iain Armstrong, Athanasios Charalampopoulos, Charlie A. Elliot, Abdul Hameed, Neil Hamilton, Judith A. Hurdman, Allan Lawrie, Jennifer T. Middleton, Hamza Zafar, Alex M. K. Rothman, Robin Condliffe, Robert A. Lewis, David G. Kiely, A. A. Roger Thompson

**Affiliations:** ^1^ Division of Clinical Medicine School of Medicine and Population Health, University of Sheffield Sheffield UK; ^2^ Sheffield Pulmonary Vascular Disease Unit Royal Hallamshire Hospital, Sheffield Teaching Hospitals NHS Foundation Trust Sheffield UK; ^3^ National Institute for Health and Care Research Sheffield Biomedical Research Centre, Sheffield Teaching Hospitals Sheffield UK; ^4^ Clinical Biochemistry, Royal Hallamshire Hospital, Sheffield Teaching Hospitals NHS Foundation Trust Sheffield UK; ^5^ Clinical Chemistry, Barnsley Hospital NHS Foundation Trust Barnsley UK; ^6^ National Heart and Lung Institute, Imperial College London London UK; ^7^ Department of Respiratory Medicine Middlemore Hospital Auckland New Zealand

**Keywords:** natriuretic peptides, point‐of‐care testing, pulmonary hypertension

## Abstract

**Background and Objectives:**

Brain natriuretic peptide (BNP) and N‐terminal prohormone of BNP (NT‐proBNP) are important biomarkers in pulmonary arterial hypertension (PAH). However, results are rarely available at the time of clinical assessment. The reliability of NT‐proBNP/BNP point‐of‐care tests (POCT) in PAH patients and the stability of NT‐proBNP in posted blood samples, to simulate remote monitoring, was investigated.

**Methods:**

Group 1 PAH patients were prospectively recruited. A sample of 40 was required to demonstrate an intraclass correlation coefficient (ICC) of 0.94 with a 95% confidence interval width of < 0.1 for agreement between POCT and the laboratory standard. Blood samples were taken at two time‐points for laboratory and POCT NT‐proBNP/BNP. Separate samples were returned to the laboratory by post and some samples were assessed pre‐ and post‐exercise assessing the impact of exercise.

**Results:**

Forty‐one patients were enrolled with 56 study visits. NT‐proBNP laboratory and POCT (*n* = 50) provided equivalent test results (Passing–Bablok slope = 1.08, CI = 0.97–1.19, intercept = 18.22, CI = −41.6 to 4.5) and ICC = 0.97. However, laboratory and POCT BNP (*n* = 49), showed non‐equivalence (Passing–Bablok slope = 1.24, CI 1.11–1.31, intercept = −5.11, CI = −9.4 to −0.46), ICC = 0.96. POCT NT‐proBNP/BNP correctly classified 92% and 86% of cases, respectively against COMPERA 2.0 4‐risk‐strata thresholds. NT‐proBNP postal laboratory samples and immediately processed NT‐proBNP laboratory samples showed good agreement and exercise had no clinically significant effect on NT‐proBNP/BNP results. Laboratory BNP identified fewer patients as high risk compared to NT‐proBNP. BNP and NT‐proBNP risk status agreed at only 57% of visits (*p* < 0.0009).

**Conclusions:**

These data support the use of POCT NT‐proBNP as a rapidly accessible and reliable alternative in clinical settings and highlight the potential of NT‐proBNP for remote monitoring via posted samples.

**Trial Registration:**

ClinicalTrials.gov registration: NCT05421949

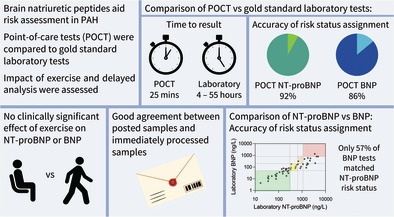

## Graphical abstract

Brain natriuretic peptides aid risk assessment in pulmonary arterial hypertension. Point‐of‐care tests for BNP and NT‐proBNP delivered fast, reliable results. Posted samples showed repeatable NT‐proBNP levels, suggesting potential for remote monitoring.

## 1 Introduction

N‐terminal Pro Hormone of BNP (NT‐proBNP) and B‐type Natriuretic Peptide (BNP) are important prognostic biomarkers in pulmonary arterial hypertension (PAH) [1, 2, 3]. NT‐proBNP and BNP are primarily released from cardiomyocytes in response to mechanical load and ventricular wall stress [4]. In PAH, rising pulmonary artery pressures lead to dysfunction of the right ventricle (RV), right heart failure, and death [5]. Right ventricular function is thought to be the primary determinant of survival in PAH. Cardiac MRI can be used to aid risk stratification, predict clinical worsening and prognosis [6, 7], but is costly and is challenging to perform remotely. Echocardiography is also a vital tool for assessment of PAH patients but requires significant operator expertise [8]. Therefore, biomarker availability is important, and NT‐proBNP and BNP have been used as markers of RV function and prognosis [9, 10].

Treatment options for PAH have expanded in the last decade, leading to improved prognosis, and categorising patients according to risk of deterioration informs initial treatment strategy and subsequent timing of treatment escalation [11, 12]. Several risk stratification tools are commonly used in categorising patients with PAH, including the 2022 ERS/ESC risk stratification, COMPERA 2.0, REVEAL 2.0, and REVEAL 2.0 Lite [13, 14, 15, 16, 17]. Integral to all is the inclusion of NT‐proBNP or BNP.

NT‐proBNP and BNP samples are collected by venepuncture, and the results may not become available to clinicians while assessing patients in the clinic. Point‐of‐care testing (POCT) for both BNP and NT‐proBNP has been established in the setting of left heart disease and reduces time to results, with potential to impact treatment decisions [4]. However, information on use of POCT for NT‐proBNP and BNP in the setting of PAH is lacking. There are also limited repeatability data for laboratory and POCT measurements of NT‐proBNP and BNP in PAH. Furthermore, no data are available to assess the reliability of POCT across the wide range of values encompassed by PAH risk stratification tools. As a result, further research is needed to investigate the reliability of POCT results associated with various threshold levels in the context of PAH.

Patients with PAH frequently travel long distances to pulmonary hypertension referral centres for face‐to‐face assessments. During the COVID‐19 pandemic, remote assessment frequently replaced face‐to‐face assessment and identified the need for objective methods for remote monitoring of PAH [18, 19]. As NT‐proBNP provides a reflection of RV dysfunction and is more stable than BNP [20], it could have a role in remote monitoring of patients with PAH and would complement remote assessment of exercise capacity. However, there are limited data on factors that may influence NT‐proBNP levels in a remote setting, such as pre‐processing time delay and exercise. The first aim of this study was to examine the agreement between POCT and laboratory NT‐proBNP and BNP across the spectrum of COMPERA 2.0 risk stratification values. Secondly, we aimed to examine the stability of NT‐proBNP as a potential tool for remote monitoring of PAH by examining the effects of delayed analysis and exercise.

## 2 Methods

Patients with Group 1 PAH were included, except for PAH‐congenital heart disease (PAH‐CHD). PAH was defined as mean pulmonary artery pressure (mPAP) > 20 mmHg, pulmonary artery wedge pressure ≤ 15 mmHg and pulmonary vascular resistance (PVR) > 240 dyn s cm^−5^ (3 Wood units). Patients had undergone systematic evaluation at a single centre, including multimodality imaging as previously described [21]. Those with a creatinine clearance of less than < 15 mL/min/m^2^ were excluded. Patients attending outpatient clinics gave informed consent to participate in the study, which was an approved sub‐study of The Sheffield Teaching Hospitals Observational Study of Patients with Pulmonary Hypertension, Cardiovascular and other Respiratory Diseases (STH‐ObS) research tissue bank (NHS Health Research Authority, Yorkshire & The Humber—Sheffield Research Ethics Committee, reference 18/YH/0441, 24/YH/0017, IRAS 248890, HTA Licence No. 12182).

### 2.1 Study Design

**FIGURE 1:**
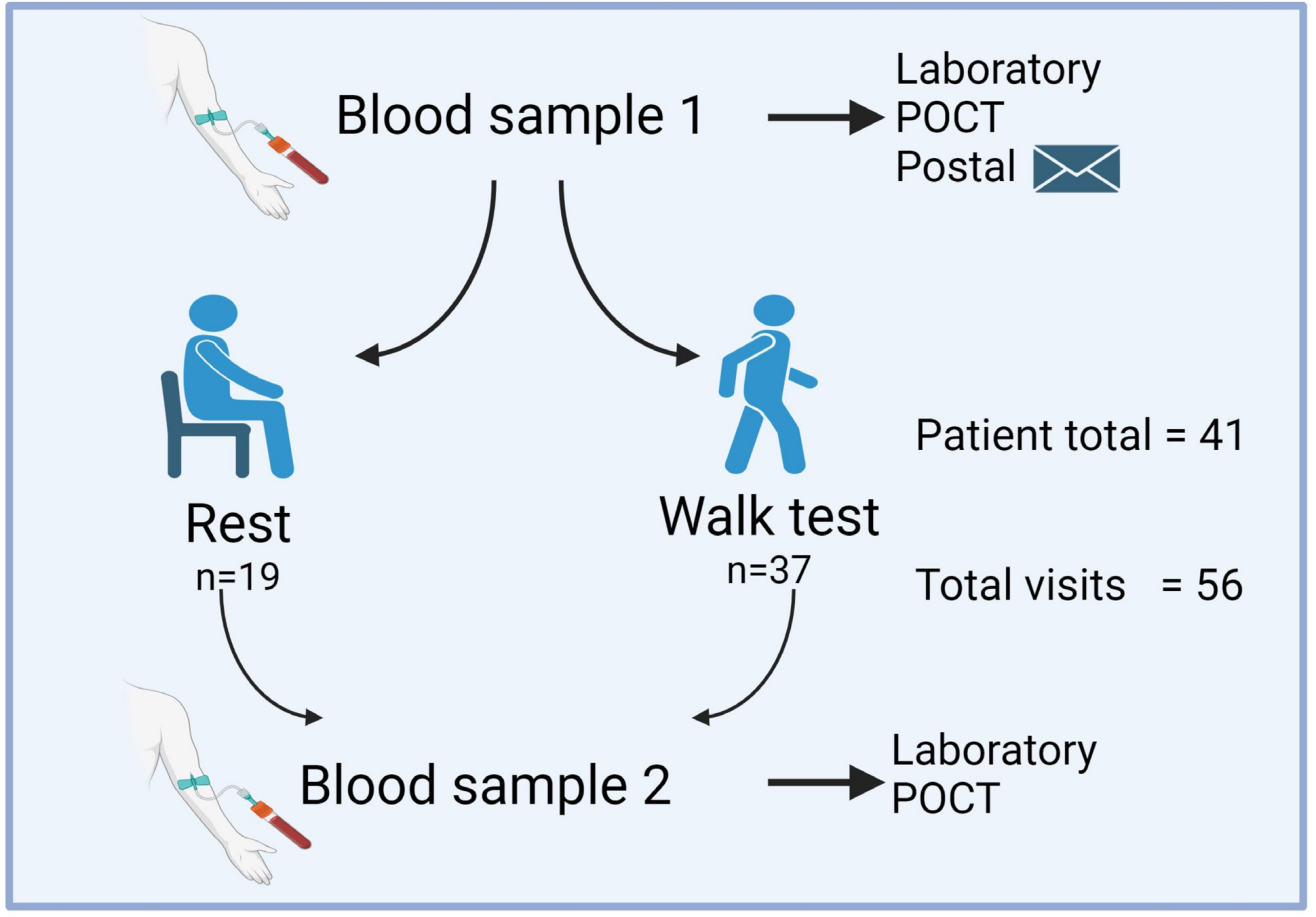
Study protocol. There were 56 patient visits, with 26 participants attending for one visit and 15 attending for two visits. Blood was taken before and after a period of rest, or before and after an incremental shuttle walk test, with samples analysed in a laboratory or by POCT as indicated. BNP, B‐type natriuretic peptide; NT‐proBNP, N‐terminal Pro Hormone of B‐type Natriuretic Peptide; POCT, point‐of‐care test. Image created in Biorender.

The study design is summarised in Figure 1. To minimise inconvenience, participants were permitted to choose between the rest or exercise group at their first visit in the study. All patients completed an initial rest period of 15 min before the first venous blood sample was taken. The exercise group then proceeded to an incremental shuttle walking test (ISWT) and the rest group were asked to sit and rest for 60 min. The second venous blood sample was taken immediately following completion of the ISWT or the rest period. A proportion of patients reattended for a second visit and were assigned to the alternative group (exercise or rest) from their first visit. Minimum sample size was determined a priori using a power calculation for intraclass correlation coefficient (ICC), with 40 individuals required to demonstrate an ICC of 0.94 with 95% confidence interval width of less than 0.1 [22].

### 2.2 POC Testing Using the Quidel Triage MeterPro

The Triage MeterPro is a widely available device for POC testing and has been used in the context of heart failure [23, 24]. A two‐level (high/low) sample quality control (QC) was performed for every new batch of 25 kits and every 30 days. An internal device quality assurance was performed before use of each test kit. Test kits were stored between 4°C and 8°C and allowed to reach room temperature prior to use. POCT was performed on whole blood drawn into blood bottles containing ethylenediaminetetraacetic acid (ETDA) (BD Biosciences). Blood was aspirated into a transfer pipette and dispensed into the sample port of the POCT test kits. Test kits for NT‐proBNP and BNP were inserted into the MeterPro device for analysis.

### 2.3 Laboratory Testing of NT‐proBNP and BNP

NHS laboratory NT‐proBNP analysis was performed on serum (BD Vacutainer, SSTII advance) using a Roche COBAS 8000 (c702) system. Aliquots of EDTA plasma were immediately frozen at −80°C for BNP analysis, as a single batch, using a Roche COBAS 8000 modular analyser system.

### 2.4 Postal Laboratory Testing for NT‐proBNP

Whole blood collected into serum tubes was either immediately delivered to the clinical chemistry laboratory or packaged in secure UN3373 compliant packaging and mailed back via external postal service. Upon receipt, NT‐proBNP level was analysed using a Roche COBAS 8000 system.

### 2.5 Incremental Shuttle Walking Test

Incremental shuttle walking tests was undertaken as previously described [25]. Patients were asked to complete a 10‐m length keeping in time with an audible bleep. Level one consists of three lengths (30 m), and each subsequent level added one extra length to the preceding level.

### 2.6 Risk Stratification

Thresholds from the COMPERA 2.0 4‐strata risk assessment tool were used to define low (BNP < 50 ng/L, NT‐proBNP < 300 ng/L), intermediate‐low (BNP < 50–199 ng/L, NT‐proBNP 300–649 ng/L), intermediate‐high (BNP 200–800 ng/L, NT‐proBNP 650–1100 ng/L) and high‐risk groups (BNP > 800 ng/L, NT‐proBNP > 1100 ng/L) [16].

### 2.7 Statistical Analysis

Statistical analysis was performed using IBM SPSS Statistics v26 (SPSS, Chicago, IL, USA) and MedCalc, version 19.4 (MedCalc Software, Ostend, Belgium). Parametric continuous variables were described by mean ± standard deviation (SD), and non‐parametric ones by median and inter‐quartile range (IQR). The repeatability of measurements taken 60 min apart, while the patient rested, was calculated for laboratory and POCT assays. The standard deviation of the two values was divided by the mean and multiplied by 100 to give a coefficient of variation (CV). Bland–Altman plots, Passing–Bablok regression, intraclass coefficients (ICC) and Lin's concordance correlation coefficient (CCC) were used to describe the relationship between POCT and laboratory test results. Paired t‐tests or Wilcoxon tests were used when examining the difference between two variables and Fisher's exact test was used to compare risk status assigned by NT‐proBNP versus BNP. A *p* value < 0.05 was deemed statistically significant.

## 3 Results

Between April 2021 and October 2022, 41 patients were recruited; demographic data are provided in Table 1. There was female predominance (71%) with a median age of 56 years. Most patients had idiopathic PAH (83%), with PAH in association with connective tissue disorders (12%) and heritable PAH (5%). Ten POCT results for NT‐proBNP were excluded due to one batch of kits failing the quality control process. All BNP POCT were processed successfully. One NT‐proBNP and one BNP laboratory sample result was missing. There were 56 visits (15 patients had 2 visits) within the study period with a median time between visits of 244 days (IQR 180–283). A larger proportion of patients were recruited to “exercise” (*n* = 37) in comparison to “rest” (*n* = 19). Median time taken from sample collection to NT‐proBNP result reporting by the hospital laboratory was 11 h (range 4–55).

**TABLE 1:** Patient demographics.

Patient demographics		
Total number of patients	41	
Total number of visits (rest/exercise)	56 (19/37)	
Female *n* (%)	29 (71)	
Age in years at first visit (median (range))	56 (29–83)	
WHO FC I/II/III/IV (%)	5/37/58/0	
Subtype of Pulmonary Arterial Hypertension *n* (%)	Idiopathic	34 (83)
	Connective Tissue Disease^a^	5 (12)
	Heritable	2 (5)
Co‐morbidities *n* (%)	Lung disease^b^	8 (20)
	Thromboembolism	5 (12)
	Obstructive Sleep Apnoea	5 (12)
	Thyroid Disease	5 (12)
	Hypertension	5 (12)
	Intracardiac shunt	5 (12)
	Obesity	4 (10)
RV function (preserved/mild/moderate/severe)^c^	13/15/6/7	
Renal function (eGFR stage 1/2/3a/3b)	13/19/6/3	
Risk category based on laboratory NT‐proBNP^d^ (low/intermediate‐low/intermediate‐high/high)	21/5/6/9	
Historical haemodynamic data (median (range))		
Right atrial pressure (mmHg)	7 (2–24)	
Mean pulmonary artery pressure (mmHg)	51 (25–77)	
Pulmonary capillary wedge pressure (mmHg)	10 (3–15)	
Cardiac output (L/min)	4.6 (1.8–7.8)	
Pulmonary vascular resistance (dynes s cm^−5^)	693 (242–2133)	

Abbreviations: RV, right ventricle; WHO FC, World Health Organisation Functional Class.

^a^ Asthma 10%, COPD 7%, bronchiectasis 2%.

^b^ Systemic sclerosis 10%, dermatomyositis 2%.

^c^ RV function assessed by echocardiogram or cardiac MRI—data from tests prior to study visit.

^d^ Based on COMPERA 2.0 four‐stratum NT‐proBNP thresholds.

### 3.1 Intra‐Assay Variability

For laboratory samples, the CV was 4.7% (*n* = 18) for NT‐proBNP and 8.7% (*n* = 19) for BNP samples, while for POCT the CV was 9.7% (*n* = 19) for NT‐proBNP and 9.2% (*n* = 19) for BNP. In addition, there was a strong correlation between NT‐proBNP and BNP (r^2^ = 0.95, *p* = < 0.001) (Supporting Information Figure 1).

### 3.2 Performance of POCT vs. Laboratory NT‐proBNP

**FIGURE 2:**
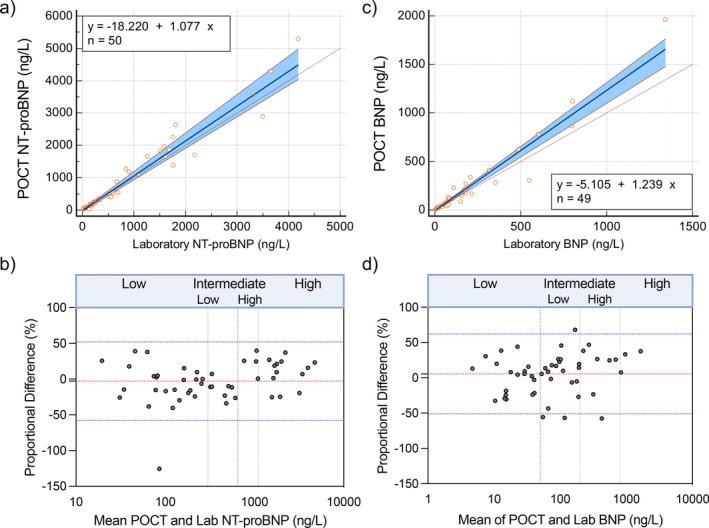
Performance of POCT versus laboratory NT‐proBNP and BNP. (a) Passing–Bablok regression for NT‐proBNP (slope = 1.08, 95% CI 0.97–1.19, intercept = −18.22, 95% CI −41.6 to 4.5; *n* = 50). (b) Bland–Altman, mean bias = −2.87 ± 27.98%, limits of agreement of −57.7% to −51.95%. (c) Passing–Bablok regression for BNP (slope = 1.24, 95% CI 1.11–1.31, intercept = −5.11, 95% CI −9.4 to −0.46; *n* = 49). (d) Bland–Altman, mean bias 5.4% ± 28.86%, limits of agreement −51.24% to 62.04%. BNP, B‐type natriuretic peptide; NT‐proBNP, N‐terminal Pro Hormone of B‐type Natriuretic Peptide; POCT, point‐of‐care test.

The relationship between POCT and laboratory NT‐proBNP results (*n* = 50) revealed an ICC of 0.97 (95% CI 0.95–0.98). Passing–Bablok regression demonstrated an estimated slope of 1.08 (95% CI 0.97–1.19), intercept of −18.22 (95% CI −41.6 to 4.5) indicating measures were equivalent (Figure 2a). When examining the agreement between absolute values of POCT and laboratory NT‐proBNP, there was evidence of proportional bias (Supporting Information Figure 2a). Therefore, Bland–Altman plots were constructed using percentage differences (Figure 2b), and this displayed a mean bias of −2.87% ± 27.98%, and limits of agreement of 57.7% to −51.95%. Lin's concordance correlation coefficient (Lin's CCC) was 0.97 (95% CI = 0.95–0.98) indicating ‘substantial’ concordance (Table 2). The performance of NT‐proBNP POCT was examined against the COMPERA 2.0 4‐strata risk scoring tool, dividing 1‐year mortality risk into low/intermediate‐low/intermediate‐high/high. NT‐proBNP POCT identified 92% patients into the correct risk category, based on the corresponding reference laboratory NT‐proBNP. Of the patients identified in the incorrect risk category, NT‐proBNP POCT classified one patient in a lower risk group (intermediate‐low vs. intermediate‐high) and three patients in a higher risk group.

**TABLE 2:** Summary table of statistics for laboratory versus POCT for NT‐proBNP and BNP.

Statistical test		POCT vs. laboratory NT‐proBNP, *n* = 50		POCT vs. laboratory BNP, *n* = 49	
Passing–Bablok regression	Slope	1.08	95% CI 0.97–1.19	1.24	95% CI 1.11–1.31
	Intercept	−18.22	95% CI −41.6 to 4.5	−5.11	95% CI −9.4 to −0.46
Bland–Altman	Mean bias	−2.87		5.4	
	SD of bias	27.98		28.86	
	Limits of agreement	−57.7 to 51.95		−51.24 to 62.04	
Intra‐class coefficient		0.98	95% CI 0.97–0.99	0.96	95% CI 0.94–0.98
Lin's concordance coefficient		0.98	95% CI 0.97–0.99	0.93	95% CI 0.90–0.95

### 3.3 Performance of POCT vs. Laboratory BNP

The agreement between POCT and laboratory BNP (*n* = 49), was not equivalent as the 95% confidence intervals for Passing–Bablok slope did not include 1 (Figure 2c, slope 1.24 [95% CI 1.11–1.31], intercept −5.11 [95% CI −9.4 to −0.46]). The Bland–Altman plot of percentage difference demonstrated a mean bias of 5.4% ± 28.86%, with limits of agreement −51.24% to 62.04% (Figure 2d). Examining the agreement between absolute values of POCT and laboratory BNP, there was evidence of proportional bias (Supporting Information Figure 2b). Lin's CCC was 0.93 (95% CI 0.90–0.95) demonstrating moderate concordance and the ICC was 0.96 (95% CI = 0.94–0.98) (Table 2). BNP POCT identified 86% of patients into the correct COMPERA 2.0 4‐strata risk group. Of those incorrectly identified, 5 were assigned in a higher risk with 2 in a lower risk group.

### 3.4 Stability of NT‐proBNP Samples Sent by Post

Of the 56 laboratory NT‐proBNP samples packaged and sent by post, 88% of samples were processed. Five samples did not return to the laboratory and 1 sample was returned but discarded in error. Postal samples were received by the laboratory 2.04 (IQR 2.35) days after being sampled. Of the remaining laboratory NT‐proBNP postal samples, 48 of 50 were analysed, 2 samples did not have a corresponding reference laboratory NT‐proBNP sample to allow comparison.

**FIGURE 3:**
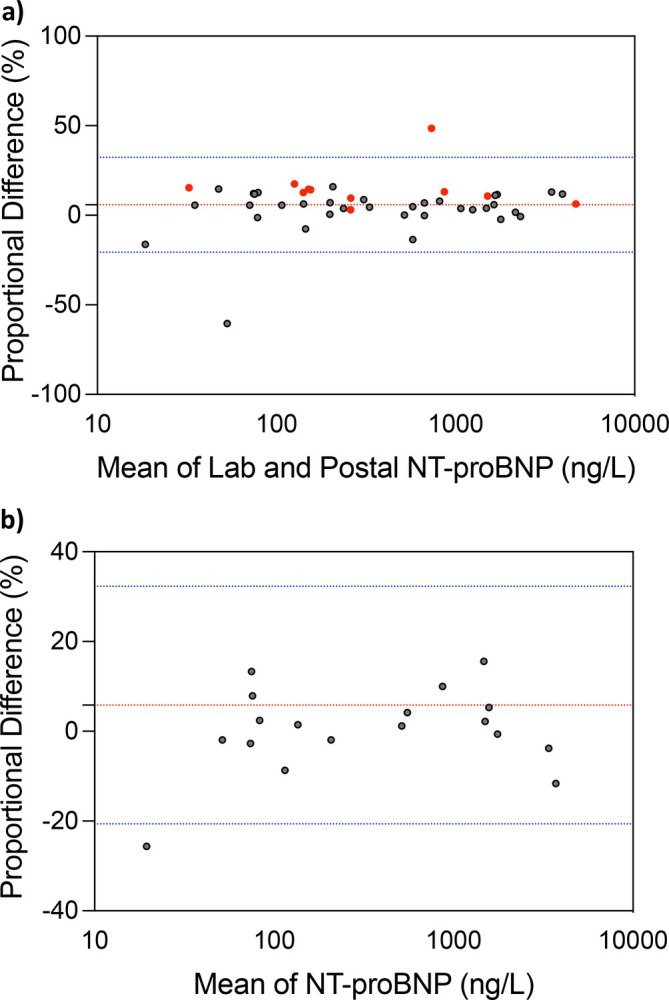
Performance of delayed NT‐proBNP laboratory analysis (posted samples) and repeated samples from rested individuals. (a) Bland–Altman demonstrating the agreement between immediately processed laboratory NT‐proBNP and postal laboratory NT‐proBNP, mean difference = 5.83% ± 13.51%, limits of agreement 32.3% to −20.64% (highlighted red points are those processed > 4 days) (*n* = 49). (b) Bland–Altman for laboratory NT‐proBNP taken twice on the same day, after resting for 1 h (*n* = 18), mean = 0.37% ± 9.51%, limits of agreement 19.01% to −18.2%.

Assessment of agreement between reference NT‐proBNP and postal NT‐proBNP samples (*n* = 48) demonstrated a mean bias of 5.83% ± 13.51% with limits of agreement −20.64% to 32.3% (Figure 3a). This is in comparison to agreement between initial laboratory NT‐proBNP and repeated laboratory NT‐proBNP (*n* = 18) which demonstrated a mean bias of 0.37% ± 9.51% with limits of agreement −18.27% to 19.01% (Figure 3b). There was a trend towards increasing proportional difference between results as the time delay to processing increased although this did not reach statistical significance (*R* ^2^ = 0.186, *p* = 0.09). Results remained accurate with less than 20% proportional difference up to a pre‐processing time delay of 6 days, although the low number of postal NT‐proBNP samples returned after 5 days limits our ability to draw firm conclusions (Supporting Information Figure 3).

### 3.5 Exercise as an Influencing Factor on NT‐proBNP

Thirty‐seven patients performed an ISWT with pre‐ and post‐exercise laboratory NT‐proBNP and 36 patients who had pre‐ and post‐exercise BNP. Paired analysis revealed a significant effect of exercise on NT‐proBNP (*p* = 0.031), the median difference was 7 ng/L. The effect of exercise on BNP was not significant (Supporting Information Figure 4a,b). In a separate cohort of 10 PAH patients, we found no change in NT‐proBNP sampled 1 h after ISWT (data not shown). There was a weak linear relationship demonstrated between ISWT and baseline NT‐proBNP (*p* = 0.008) and baseline BNP (*p* = 0.004) (Supporting Information Figure 5a,b) and there was also no correlation between distance walked on ISWT and % change in NT‐proBNP or BNP values following exercise (Supporting Information Figure 5c,d).

### 3.6 Comparison of NT‐proBNP and BNP and Assignment of Risk Status

**FIGURE 4:**
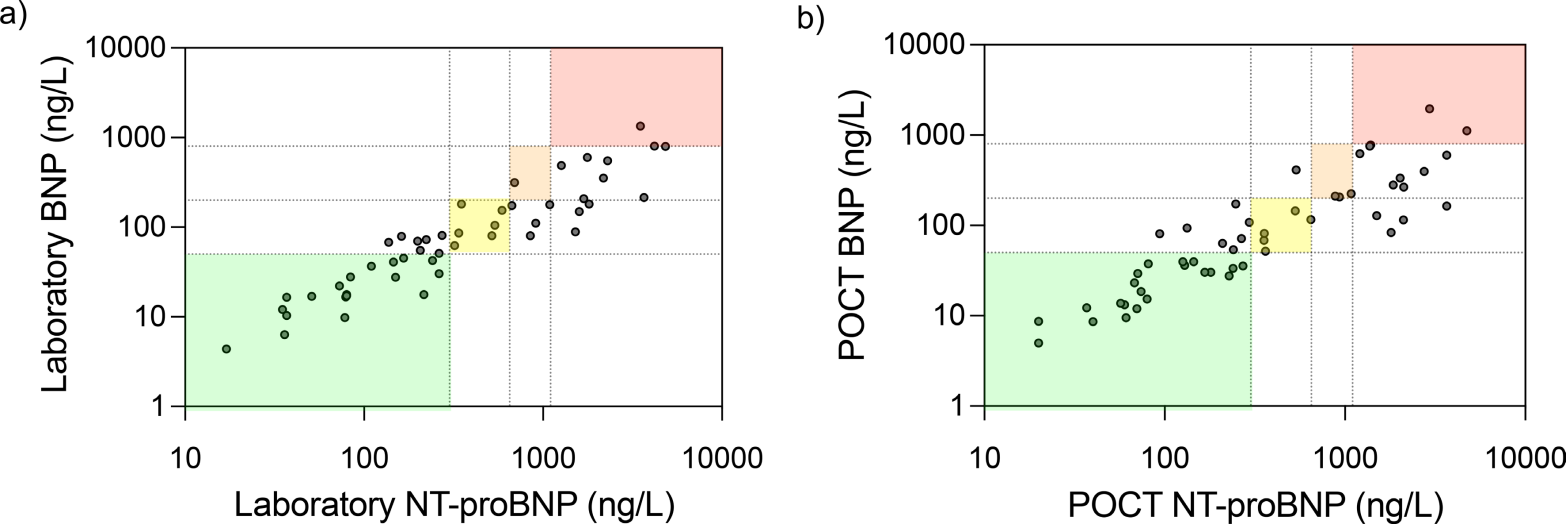
Comparisons of risk status assignment by NT‐proBNP and BNP (laboratory and POCT). Scatter plots showing the relationship between BNP and NT‐proBNP measured by (a) hospital laboratory or (b) POCT. COMPERA 2.0 4‐strata risk thresholds are indicated by dashed lines and shaded segments indicate matching risk status. BNP, B‐type natriuretic peptide; NT‐proBNP, N‐terminal Pro Hormone of B‐type Natriuretic Peptide; POCT, point‐of‐care test.

NT‐proBNP was strongly correlated with BNP (Spearman *r* = 0.945 (laboratory tests, *n* = 49) and *r* = 0.905 (POCT, *n* = 51), both *p* < 0.0001). However, fewer patients had BNP values above the high‐risk threshold of 800 ng/L (*n* = 2) compared to the number above the NT‐proBNP threshold of 1100 ng/L (*n* = 12) (Figure 4a,b). BNP and NT‐proBNP risk status agreed in only 57% (28/49) of laboratory paired samples and 60% (31/51) of POCT paired samples. The differences in patient risk status assigned across the COMPERA 2.0 4‐strata were statistically significant (Fisher's exact test *p* = 0.0007 laboratory, *p* = 0.0001 POCT).

## 4 Discussion

This study provides insights into the reliability of near‐patient tests of NT‐proBNP and BNP and demonstrates potential for NT‐proBNP to be used to remotely monitor patients with PAH. POCT for NT‐proBNP correctly identified the patients' risk category in a higher proportion of patients than BNP, suggesting NT‐proBNP POCT is the more reliable tool in the setting of PAH. We also observed that a pre‐processing time delay did not significantly alter NT‐proBNP laboratory results for samples returned within 5 days of sampling.

### 4.1 Point‐of‐Care NT‐proBNP/BNP and Laboratory NT‐proBNP/BNP

The first aim of this study was to assess the relationship between POCT and laboratory measurements of NT‐proBNP and BNP. Several statistical methods were utilised. For NT‐proBNP laboratory versus NT‐proBNP POCT, there was evidence of proportional bias, and Bland–Altman plots using percentage differences demonstrated wide limits of agreement; however, Passing–Bablok deemed the two assays to be equivalent. ICC was in the excellent range, and Lin's CCC showed substantial concordance. Despite the proportional bias, when examined against the COMPERA 2.0 4‐strata risk tool, 92% of POCT results were in the correct risk grouping according to their laboratory comparator.

For BNP laboratory versus BNP POCT, Bland–Altman analysis showed that limits of agreement were wide and the Passing–Bablok was not equivalent. ICC was high, however Lin's CCC demonstrated moderate concordance. BNP POCT correctly classified COMPERA 2.0 4‐strata risk group in 86% of patients. While limits of agreement for POCT versus laboratory tests were over 50%, in practical terms both NT‐proBNP and BNP POCT results identified correct risk category for the majority.

The major benefit for utilising NT‐proBNP and BNP POCT in clinical practice is the shorter processing time compared to formal laboratory analysis. Processing time for laboratory NT‐proBNP and BNP will be dependent upon the laboratory, and we found a median time of 11 h for NT‐proBNP in this study. In contrast, POCT for NT‐proBNP and BNP, provided results in approximately 25 min. Laboratory testing of NT‐proBNP and BNP is complex, requires specialist equipment and trained laboratory staff [26]. Multiple studies have explored the ease of processing POCT. In one study, after 2 training sessions (1.5 h), GPs were successfully taught to use and interpret BNP POCT in the setting of left heart failure [24]. In another “untrained user study” using POCT BNP, standard user instructions were provided to operators and the coefficient of variation obtained was similar to that reported in studies published by clinicians experienced with the same device [27]. BNP POCT assays are already established in the screening of patients presenting with unexplained breathlessness and for heart failure [23].

An important consideration is the cost of NT‐proBNP and BNP POCT in comparison to laboratory processing. The National Institute for Health and Care Excellence (NICE) estimates the cost of laboratory NT‐proBNP/BNP to be 15–25 GBP. In comparison, it costs approximately 29 GBP per test kit to process each POCT NT‐proBNP/BNP. Initial purchase cost, maintenance cost, and the staff time for training and processing need to be considered for both laboratory and POCT.

### 4.2 Remote Monitoring

Remote monitoring of liver function tests for patients prescribed endothelin receptor antagonists, is well‐established in PH [28]. Patients are sent a pre‐labelled box and equipment, and are asked to attend their local phlebotomy service [29]. To our knowledge remote monitoring for laboratory NT‐proBNP has not been previously explored. In our analysis we found that agreement was similar between the delayed postal laboratory samples in comparison to the immediately processed laboratory samples. Eighty‐eight percent of samples were returned to the laboratory and processed without issue. It was noted there were two anomalous values, one returned at 2 days and the other 7 days. We also noted increasing variability of results with longer time delay (Supporting Information Figure 3), highlighting that longer delays in analysis could potentially impact on results. This study did not attempt to control for factors such as sampling time or temperature, to assess performance in a real‐world setting. However, we did assess the effect of exercise prior to sampling.

### 4.3 Effects of Exercise on NT‐proBNP and BNP

There are limited data on exercise and its impact on NT‐proBNP and BNP results. There are convincing data from a pooled systematic review of 27 studies to suggest that in patients with heart failure, rehabilitation exercise programmes comprising several sessions over a sustained period lower NT‐proBNP and BNP levels, implying improved heart function [30]. However, there are limited data examining shorter bursts of exercise in patients with heart failure or PAH. One study examined BNP in 13 patients with stable heart failure. Patients either performed an endurance cycle for 30 min or a high intensity training (HIT) session. BNP was increased in both types of exercise, but more so immediately following the HIT session and fell at 2 h to baseline. For the endurance cycle, there was a rise immediately following cessation of exercise, but BNP continued to rise 2 h following cessation of exercise [31]. A further study demonstrated a rise in NT‐proBNP at maximum exercise in all but one patient of 20 PAH patients with PAH, indicating NT‐proBNP may have an immediate rise with exertion [32]. NT‐proBNP was also examined in 63 therapy‐naïve PAH patients, who demonstrated a rise in level from baseline to peak exercise [33]. In our study, we examined 37 patients who underwent an ISWT, which is regarded as a maximal test. Laboratory NT‐proBNP and BNP were sampled prior to the ISWT and immediately after to provide a comparison. We found a significant rise in laboratory NT‐proBNP (*p* = 0.031); however, the median difference was only 7 ng/L and unlikely to be clinically significant. In contrast, no significant difference was noted in BNP after exercise. It may be difficult to control conditions when patients have blood sampled remotely, but our results suggest that exercise around the time sampling is unlikely to increase NT‐proBNP or BNP levels such that it would impact on decision making in PAH patients.

### 4.4 Comparison of NT‐proBNP and BNP

There were significant differences in COMPERA 2.0 4‐strata risk status assigned by NT‐proBNP or BNP, whether POCT or laboratory results were used. The BNP threshold for high risk identified only 2 patients in this small cohort versus 12 identified by the NT‐proBNP threshold. This could imply that BNP underestimated risk status in our study population, or that NT‐proBNP overestimated risk. There were insufficient data to propose new thresholds, but this observation could have significant clinical implications and merits further investigation.

### 4.5 Limitations

This study was limited by its inclusion of only PAH patients at a single centre, however it would be unlikely for samples from patients with other forms of PH, such as chronic thromboembolic pulmonary hypertension, to alter assay performance. We also tested only one globally available POCT device. Although we observed no clinically significant change in NT‐proBNP or BNP levels following exercise it is possible that more prolonged exercise may have altered the levels. However, our data suggest that daily activities and standard field walking tests are unlikely to have a clinically significant impact on NT‐proBNP and BNP levels. For POCT, several NT‐proBNP POCT test kits failed the quality control process due to temporary problems with kit temperature regulation, highlighting that POCT test kits are temperature sensitive, and care must be taken to maintain correct storage of equipment. Twelve percent of postal NT‐proBNP samples were not processed because they were ‘lost’ in the post or had been mistakenly discarded. Tracking of returned samples would be important in the clinical application of remote monitoring, as would further work assessing the impact of environmental conditions on blood samples in transit. The study was not powered or designed to assess impact of result availability on management or to assess other factors that might impact natriuretic peptide level, for example renal function.

POCT can provide an alternative to laboratory testing and has the added value of being quick and easy to process and available at the time of the clinical consultation. Cost may limit use in some healthcare settings. Postal laboratory NT‐proBNP samples provide reliable results highlighting that NT‐proBNP could be incorporated into remote clinical assessments.

## Author Contributions

**Charlotte Durrington:** conceptualization (lead), data curation (lead), formal analysis (lead), investigation (equal), methodology (lead), writing – original draft (lead), writing – review and editing (lead). **Christian Battersby:** data curation (supporting), investigation (supporting), project administration (supporting). **Laura Holt:** data curation (supporting), investigation (supporting). **Alexandra Fairman:** data curation (supporting), investigation (supporting), project administration (supporting). **Scarlett Strickland:** data curation (supporting), investigation (supporting). **Thomas Salisbury:** data curation (supporting), investigation (supporting). **Helena A. Turton:** data curation (supporting), investigation (supporting). **Lisa Watson:** funding acquisition (supporting), project administration (supporting). **Ian Smith:** data curation (supporting). **Stefan Roman:** data curation (supporting), investigation (supporting). **Jenna Ablott:** data curation (supporting), investigation (supporting). **Felicity Hitchcock:** data curation (supporting), investigation (supporting). **Chloe Roddis:** data curation (supporting), investigation (supporting). **Eleanor Oakes:** formal analysis (supporting). **Heather Wilshaw:** data curation (supporting). **Iain Woodrow:** conceptualization (supporting), data curation (supporting). **Iain Armstrong:** conceptualization (supporting). **Athanasios Charalampopoulos:** conceptualization (supporting), supervision (supporting), writing – review and editing (supporting). **Charlie A. Elliot:** conceptualization (supporting), supervision (supporting), writing – review and editing (supporting). **Abdul Hameed:** conceptualization (supporting), supervision (supporting), writing – review and editing (supporting). **Neil Hamilton:** conceptualization (supporting), data curation (supporting), writing – review and editing (supporting). **Judith A. Hurdman:** conceptualization (supporting), supervision (supporting), writing – review and editing (supporting). **Allan Lawrie:** conceptualization (supporting), methodology (supporting), supervision (supporting). **Jennifer T. Middleton:** data curation (supporting). **Hamza Zafar:** data curation (supporting). **Alex M. K. Rothman:** conceptualization (supporting), methodology (supporting), supervision (supporting). **Robin Condliffe:** conceptualization (supporting), methodology (supporting), supervision (supporting), writing – review and editing (supporting). **Robert A. Lewis:** conceptualization (lead), funding acquisition (equal), methodology (lead), visualization (lead), writing – review and editing (lead). **David G. Kiely:** conceptualization (lead), formal analysis (lead), funding acquisition (equal), methodology (lead), supervision (lead), writing – original draft (lead), writing – review and editing (lead). **A. A. Roger Thompson:** conceptualization (lead), data curation (equal), formal analysis (equal), funding acquisition (equal), investigation (equal), methodology (lead), supervision (lead), visualization (lead), writing – original draft (lead), writing – review and editing (lead).

## Ethics Statement

This study was performed in accordance with the Declaration of Helsinki. This human study was approved by NHS Health Research Authority, Yorkshire & The Humber—Sheffield Research Ethics Committee—approval: reference 18/YH/0441, IRAS 248890, HTA Licence No. 12182. All adult participants provided written informed consent to participate in this study.

## Conflicts of Interest

Athanasios Charalampopoulos reports lecture honoraria from Janssen and Boehringer, outside the submitted work. Charlie A. Elliot reports lecture honoraria and travel support from Janssen Pharmaceuticals, outside the submitted work. Abdul Hameed reports lecture honoraria and travel support from Janssen, outside the submitted work. Neil Hamilton reports consulting fees and travel support from Janssen, lecture honoraria from MSD and Janssen, advisory board participation with Bayer, MSD, Janssen and Vifor, and a leadership role as pharmacist for the NHS Specialist Respiratory Clinical Reference Group, outside the submitted work. Alex M. K. Rothman reports grants from a Wellcome Trust Clinical Research Career Development Fellowship (206632/Z/17/Z), MRC (experimental medicine grant MR/W026279/1), Abbott Laboratories, Medtronic Inc., Endotronix, SoniVie, NXT Biomedical, Gradient and Neptune Medical, outside the submitted work. Robin Condliffe reports lecture honoraria, advisory boards and travel support from Janssen Pharmaceuticals and MSD, outside the submitted work. Robert A. Lewis reports grants from Janssen Pharmaceuticals. David G. Kiely reports support for the present manuscript from NIHR Sheffield Biomedical Research Centre, and also reports grants from Janssen Pharmaceuticals, NIHR Sheffield Biomedical Research Centre and Ferrer, consulting fees and lecture honoraria from Janssen Pharmaceuticals, Ferrer, Apollo, Altavant, Liquidia, MSD and United Therapeutics, travel support from Janssen, Ferrer, MSD and Unit, advisory board membership for Ferrer, Janssen, Liquidia and MSD, and leadership roles as a member of the Clinical Reference Group for Specialised Respiratory Medicine (NHS England), and lead of the UK National Audit of Pulmonary Hypertension, outside the submitted work. A. A. Roger Thompson reports grants from British Heart Foundation and NIHR, and lecture honoraria and travel support from Janssen‐Cilag Ltd., outside the submitted work. The remaining authors declare no conflicts of interest.

## Supporting information


**Data S1.** Supporting Information.

## Data Availability

The data that support the findings of this study are available on request from the corresponding author. The data are not publicly available due to privacy or ethical restrictions.
