# Supplementary material for: NT‐proBNP and BNP Testing in Pulmonary Arterial Hypertension: Point‐of‐Care and Remote Monitoring
Source: Respirology. 2025 Jul 3;30(11):1094–103. doi: 10.1111/resp.70087 (PMC12581095; doi:10.1111/resp.70087)
Supplement: Supplementary file 1 — Data S1. Supporting Information. [file RESP-30-1094-s001.docx]

**Supporting information**

**NT-proBNP and BNP testing in pulmonary arterial hypertension: point of care and remote monitoring**

Charlotte Durrington^1, 2^, Christian Battersby^1^, Laura Holt^1^, Alexandra Fairman^3^, Scarlett Strickland^1, 2, 3^, Thomas Salisbury^2^, Helena A. Turton^1^, Lisa Watson^2^, Ian Smith^2^, Stefan Roman^2^, Jenna Ablott^2^, Felicity Hitchcock^2^, Chloe Roddis^2^, Eleanor Oakes^4^, Heather Wilshaw^4^, Iain Woodrow^5^, Iain Armstrong^2^, Athanasios Charalampopoulos^2^, Charlie A. Elliot^2^, Abdul Hameed^2^, Neil Hamilton^2^, Judith A. Hurdman^2^, Allan Lawrie^6^, Jennifer T. Middleton^1,2^, Hamza Zafar^1,2^, Alex M. K. Rothman^1,2,3^, Robin Condliffe^1,2,3^, Robert A. Lewis^7^, David G. Kiely^1,2.3^, A. A. Roger Thompson^1,2,3^

1.Division of Clinical Medicine, School of Medicine and Population Health, University of Sheffield, Sheffield, UK.

2. Sheffield Pulmonary Vascular Disease Unit, Royal Hallamshire Hospital, Sheffield Teaching Hospitals NHS Foundation Trust, Sheffield, UK.

3. National Institute for Health and Care Research Sheffield Biomedical Research Centre, Sheffield Teaching Hospitals, Sheffield, UK.

4. Clinical Biochemistry, Royal Hallamshire Hospital, Sheffield Teaching Hospitals NHS Foundation Trust, Sheffield, UK.

5. Clinical Chemistry, Barnsley Hospital NHS Foundation Trust, Barnsley, UK.

6. National Heart and Lung Institute, Imperial College London, London, UK.

7. Department of Respiratory Medicine, Middlemore Hospital, Auckland, New Zealand.

**Correspondence:**

A. A. Roger Thompson, R.Thompson@sheffield.ac.uk

**Shared senior authorship:**

David G. Kiely and A. A. Roger Thompson contributed equally to this research study.

**Supplementary Figure 1**


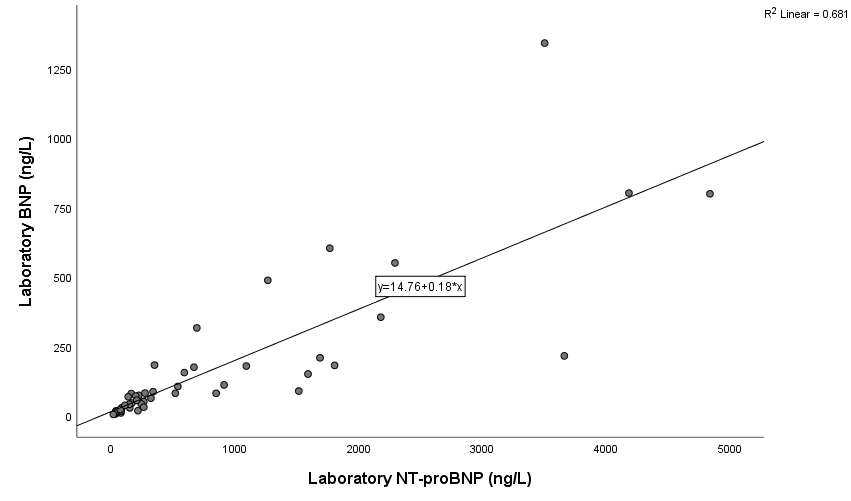


Supplementary figure 1. Spearman’s rank coefficient for laboratory NT-proBNP and BNP, r^s^ = 0.945, p=<0.01, (n=49).

**Supplementary Figure 2**

**a)**


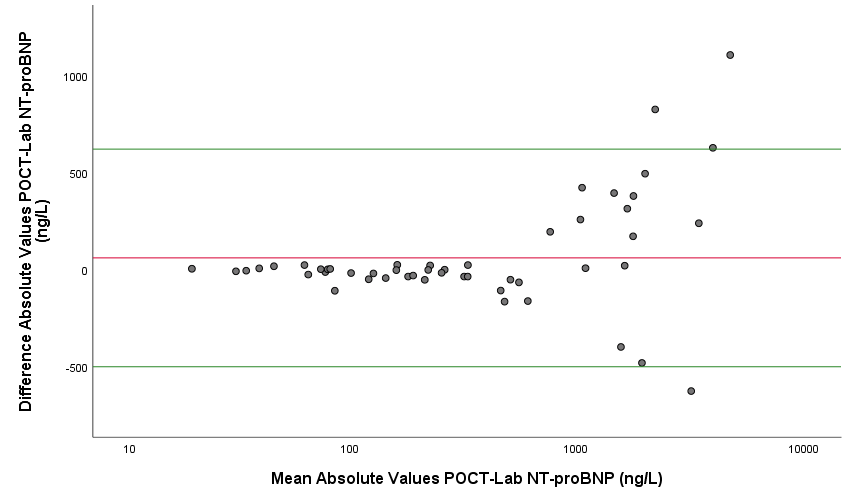
**b)**


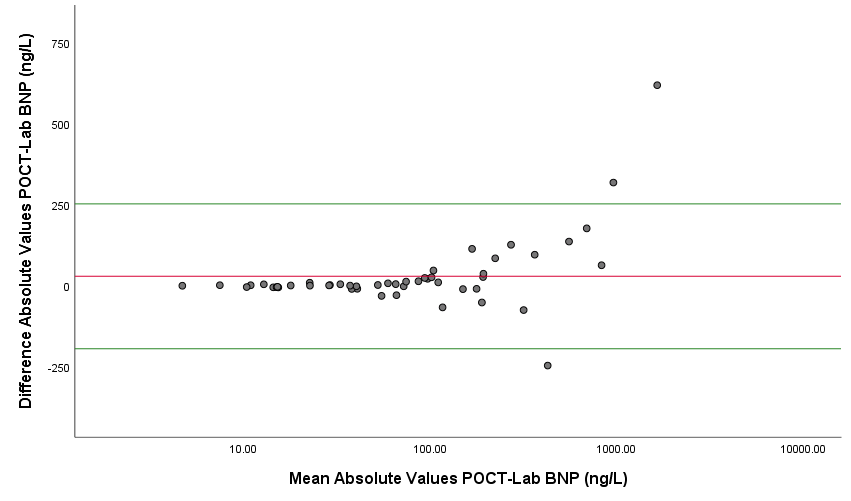
Supplementary figure 2. (a) Bland-Altman demonstrating the agreement between the absolute values of laboratory NT-proBNP, mean bias = 60.9±285.4, limits of agreement 620.3 to -498.5 (n=50). (b) Bland-Altman for laboratory BNP, mean bias = 29.8±114.01, limits of agreement 253.3 to -193.7 (n=49).

**Supplementary Figure 3**

Supplementary figure 3. Relationship between pre-processing time delay and proportional difference in NT-proBNP result.Proportional difference between immediately processed laboratory NT-proBNP and samples returned to the laboratory by post, over time (n=48).

**Supplementary Figure 4**

a)
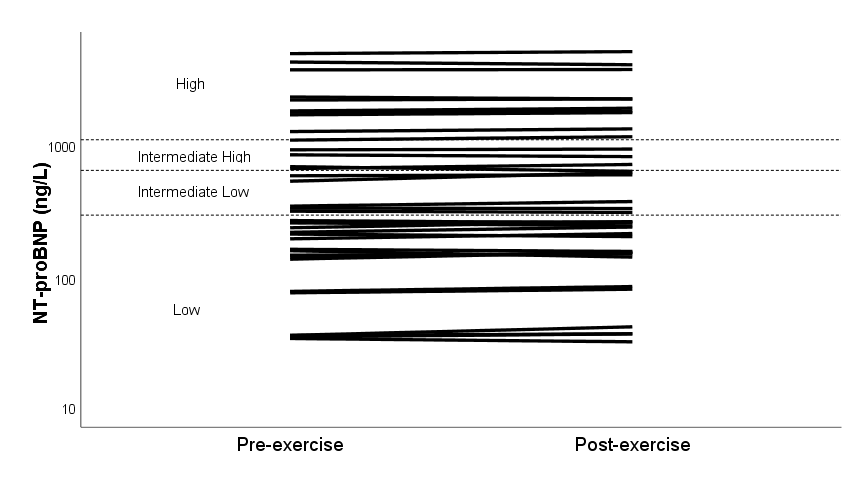


P=0.03

b)


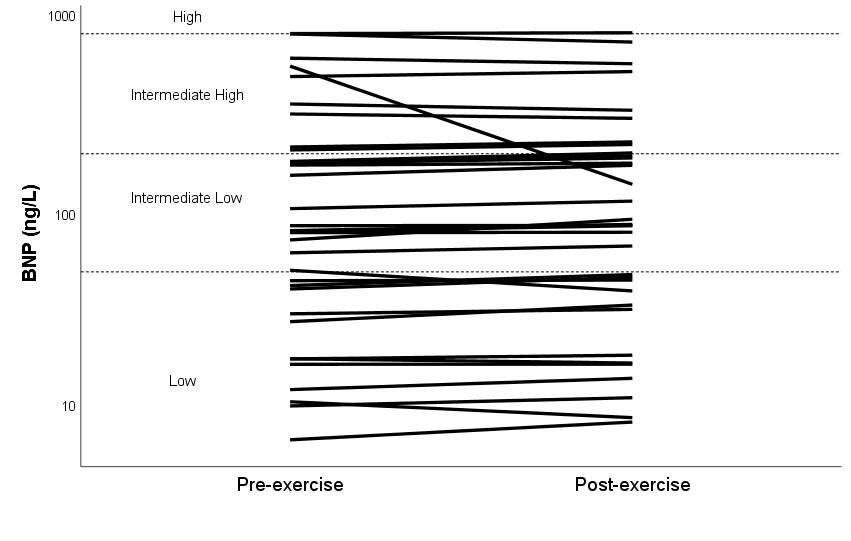


P=0.10

Supplementary figure 4. The effect of exercise (pre- and post-incremental shuttle walk test) on a) NT-proBNP, n=37 and b) BNP, n=36. Dashed lines indicate thresholds for COMPERA 2.0 4-strata risk status. Data analysed by Wilcoxon Signed Rank test.

**Supplementary Figure 5**

**a)**


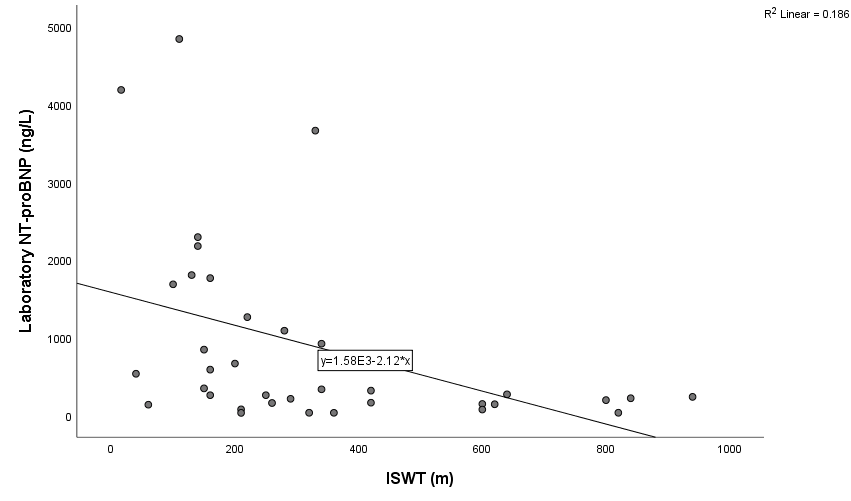


R^2^ Linear = 0.186

P=0.008

**b)**


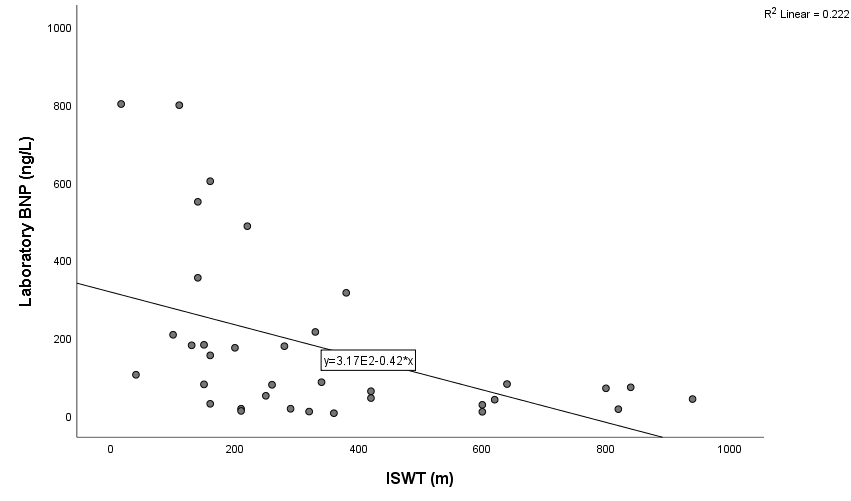


R^2^ Linear = 0.222

P=0.004

**c)**


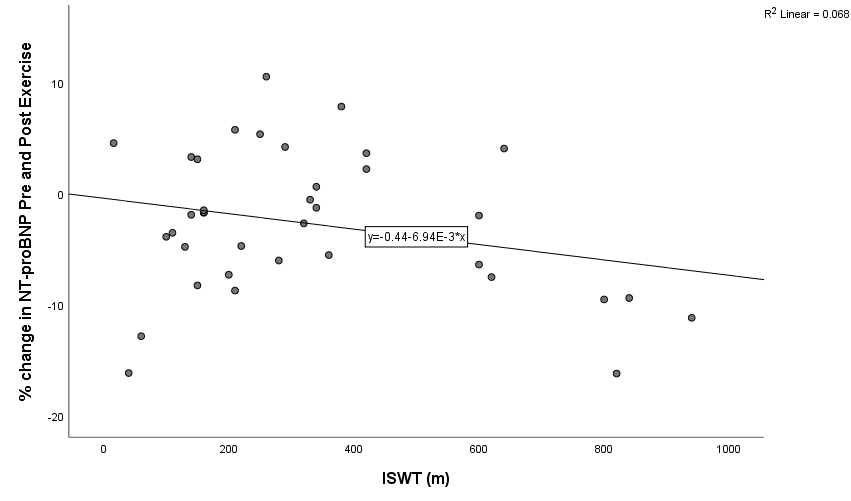


**d)**


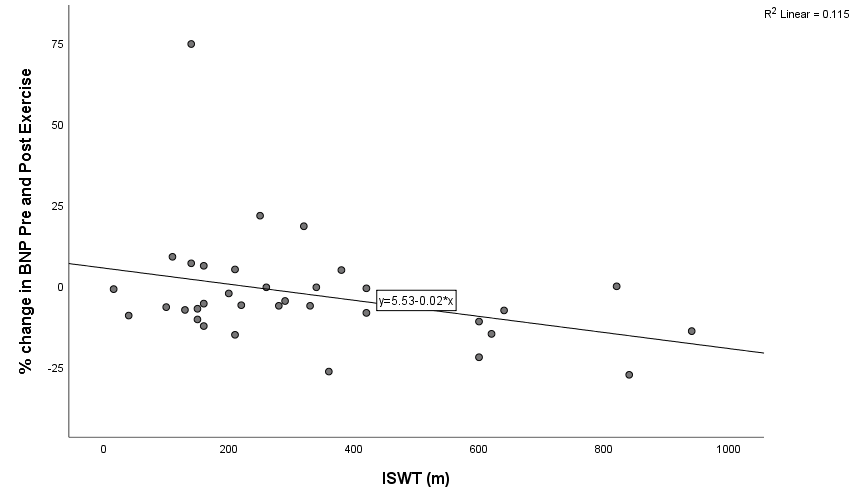


Supplementary figure 5. (a) Absolute laboratory NT-proBNP pre ISWT plotted against ISWT, (n37), (b) Absolute laboratory BNP pre ISWT plotted against ISWT, (n=36). (c) % change in laboratory NT-proBNP pre and post ISWT, (n=37), (d) % change in laboratory BNP, (n=36).
